# Distinguishing the Origin of Asteroid (16) Psyche

**DOI:** 10.1007/s11214-022-00880-9

**Published:** 2022-04-12

**Authors:** Linda T. Elkins-Tanton, Erik Asphaug, James F. Bell, Carver J. Bierson, Bruce G. Bills, William F. Bottke, Samuel W. Courville, Steven D. Dibb, Insoo Jun, David J. Lawrence, Simone Marchi, Timothy J. McCoy, Jose M. G. Merayo, Rona Oran, Joseph G. O’Rourke, Ryan S. Park, Patrick N. Peplowski, Thomas H. Prettyman, Carol A. Raymond, Benjamin P. Weiss, Mark A. Wieczorek, Maria T. Zuber

**Affiliations:** 1 School of Earth and Space Exploration, Arizona State University, Tempe, AZ 86387-2001 USA; 2 Lunar and Planetary Laboratory, University of Arizona, Tucson, AZ 85721-0092 USA; 3 Jet Propulsion Laboratory, Pasadena, CA 91109 USA; 4 Southwest Research Institute, Boulder, CO 80302 USA; 5 Johns Hopkins University Applied Physics Laboratory, Laurel, MD 20723 USA; 6 Smithsonian National Museum of Natural History, Washington, DC 20013 USA; 7 National Space Institute, Danish Technical University, Lyngby, Denmark; 8 Planetary Science Institute, Tucson, AZ 85719 USA; 9 Department of Earth, Atmospheric and Planetary Sciences, Massachusetts Institute of Technology, Cambridge, MA 02139-4307 USA; 10 Observatoire de la Côte d’Azur, CNRS, Laboratoire Lagrange, Université Côte d’Azur, Nice, France

**Keywords:** Asteroid, Psyche, Space exploration, Planetesimal, Meteorite

## Abstract

The asteroid (16) Psyche may be the metal-rich remnant of a differentiated planetesimal, or it may be a highly reduced, metal-rich asteroidal material that never differentiated. The NASA Psyche mission aims to determine Psyche’s provenance. Here we describe the possible solar system regions of origin for Psyche, prior to its likely implantation into the asteroid belt, the physical and chemical processes that can enrich metal in an asteroid, and possible meteoritic analogs. The spacecraft payload is designed to be able to discriminate among possible formation theories. The project will determine Psyche’s origin and formation by measuring any strong remanent magnetic fields, which would imply it was the core of a differentiated body; the scale of metal to silicate mixing will be determined by both the neutron spectrometers and the filtered images; the degree of disruption between metal and rock may be determined by the correlation of gravity with composition; some mineralogy (e.g., modeled silicate/metal ratio, and inferred existence of low-calcium pyroxene or olivine, for example) will be detected using filtered images; and the nickel content of Psyche’s metal phase will be measured using the GRNS.

## Introduction to Science Objective 1, Is Psyche a Core?

The first, and central, science objective for the NASA Psyche mission is to determine whether the asteroid (16) Psyche (henceforth referred to as “the asteroid Psyche”, to distinguish it from the mission itself, which is also named Psyche) is part or the whole of a planetesimal core, or, if it is not, what is its most likely provenance.

In our summary paper, Elkins-Tanton et al. (2020), we considered all data on the physical and chemical properties of Psyche. The asteroid’s bulk density appears to be between 3,400 and $4{,}100~\text{kg}\,\text{m}^{-3}$. Given the known densities of meteoritic metal ($\sim7000\text{--}8000~\text{kg/m}^{-3}$), and assuming not more than 20% porosity (see argument in Elkins-Tanton et al. 2020), the body could contain up to 60 vol.% metal. If porosities higher than expected on a body of Psyche’s size are enabled by the material properties of metals at relevant space temperatures, then Psyche’s metal fraction could be far higher than this. In particular, if Psyche consists of exclusively metal and pore space, then the volume ratio of these components must be around 50:50.

The asteroid is therefore likely a mixture of metal and an additional lower-density component or components. Based upon modeling of available telescopic and laboratory spectral characteristics (Dibb 2021), the additional components are likely to be very low in FeO or Fe_2_O_3_. Likely candidates for a non-metal fraction of the asteroid include low-iron pyroxene (e.g., Hardersen et al. 2005), sulfides (e.g., Clark et al. 2004), or perhaps carbon compounds.

**Fig. 1:**
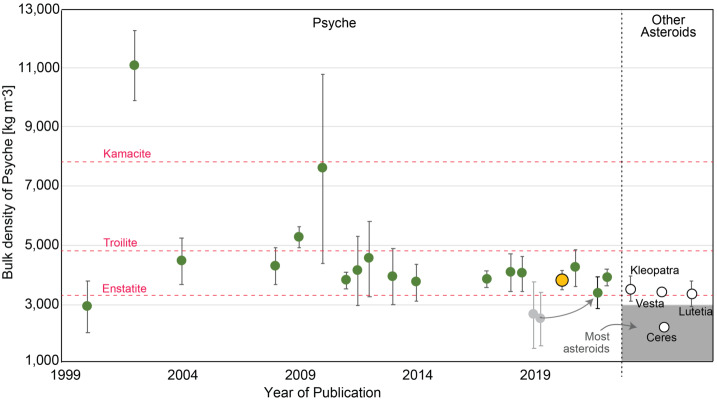
The average bulk density of Psyche assumed by this project is $3{,}780\pm340~\text{kg}\,\text{m}^{-3}$ (large yellow disk; Elkins-Tanton et al. (2020)). The lowest estimates, from Siltala and Granvik (pale grey disks) were revised upward in 2021 (gray arrow to new estimate) (Siltala and Granvik 2021). Additional data from Ferrais et al. (2020) and Shepard et al. (2021) added to the data compiled in Elkins-Tanton et al. (2020). For comparison, the densities of several minerals pertinent to formation hypotheses of Psyche are included: $7{,}870~\text{kg}\,\text{m}^{-3}$ for kamacite, $4{,}840~\text{kg}\,\text{m}^{-3}$ for troilite, and $3{,}270~\text{kg}\,\text{m}^{-3}$ for enstatite (Mg-rich pyroxene) (Bass 1995; Smyth and McCormick 1995) (pink dashed lines). For comparison, and not placed in the year of their publication asteroid densities are: Kleopatra ($3{,}380\pm500~\text{kg}\,\text{m}^{-3}$; Marchis et al. 2021), Vesta ($3{,}454~\text{kg}\,\text{m}^{-3}$; Russell et al. 2012), Lutetia ($3{,}400\pm300~\text{kg}\,\text{m}^{-3}$; Vernazza et al. 2011), and Ceres ($2{,}161~\text{kg}\,\text{m}^{-3}$; Park et al. 2019) (empty circles). The field of most asteroids’ densities from Fienga et al. (2020). Psyche remains one of the densest asteroids known

More recent observations and calculations have not changed this scenario. For example, an anomalously low density estimate of the asteroid Psyche by Siltala and Granvik (2019) was updated (Siltala and Granvik 2021), providing a value of $3{,}880~\text{kg}\,\text{m}^{-3}$. Now all recent density estimates, including Viikinkoski et al. (2018), Drummond et al. (2018), Baer and Chesley (2017), and others, falls within the range given in Elkins-Tanton et al. (2020) of 3,400 and $4{,}100~\text{kg}\,\text{m}^{-3}$ (Fig. 1).

Furthermore, Becker et al. (2020) obtained UV spectra of the asteroid Psyche using the Hubble Space Telescope, showing that the surface could be interpreted as pure metal. They also cautioned that when modeling the observed spectra using intimate mixture modeling, the model spectra are highly sensitive to the assumed grain sizes of the iron and silicate particles: spectra similar to the observations could be obtained with iron volume fractions as low as 10%.

**Fig. 2:**
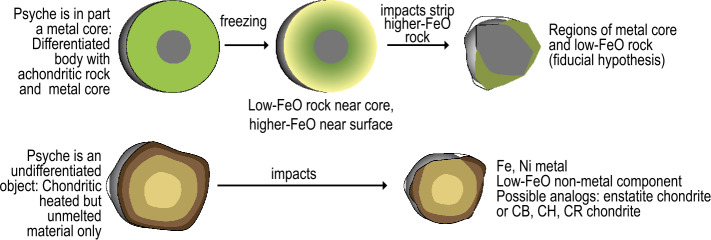
Formation hypotheses that are consistent with Psyche’s density and relatively featureless (low-FeO) spectra include a differentiated parent body (the fiducial hypothesis) and certain chondritic, undifferentiated materials (enstatite or CB-chondritic or similar low-FeO composition)

These density and compositional data provide constraints on asteroid Psyche’s possible formation. Our favored fiducial model for the asteroid’s formation is as the remains of a differentiated body containing core metal (Fig. 2). Psyche may contain a large fraction of a metal core and some silicate mantle retained on the outside, or the silicate fraction could be mixed into the metal fraction. The silicate fraction must, however, contain strikingly little iron, evidenced by the lack of strong absorption bands (for example, Hardersen et al. 2005).

Formation of such a low iron-oxide bulk rock composition seems, within the current state of planet formation theory, to require one of two processes to have occurred: (1) Silicate differentiation that produces low-iron-oxide silicates near the core and high-iron-oxides silicates nearer the surface, with subsequent removal of the outermost high-iron-oxide fraction by stripping impacts; or (2) Formation of the Psyche parent body from highly reduced primitive material in which the iron has been reduced to its metallic form and incorporated into the core, leaving the silicates naturally low in iron and high in magnesium.

Alternatively, the asteroid could be primarily composed of primitive (unmelted) high-metal material, perhaps a chondrite with silicates already low in iron-oxide, maybe even from a parent body already sampled in the terrestrial meteorite collection. We have hypothesized that Psyche could be formed from highly reduced material that formed near the Sun (Weidenschilling 1978), but we argue below that an origin beyond Jupiter might, in fact, be more likely.

When the Psyche orbiter reaches the asteroid, all its science instruments will contribute to answering this science objective. The total iron content (in metal and silicates) will be determined from element data acquired by the Gamma Ray and Neutron Spectrometer (GRNS; Lawrence et al. this journal) and from filter data from the Psyche Multispectral Imager (Bell et al. this journal). The gravity investigation (Zuber et al. this journal) will provide Psyche’s bulk density, an important additional constraint on the total iron content, and combined with images and topography will inform the search for correlations between compositional and structural information. The magnetometer (Weiss et al. this journal) will seek the signature of an original core dynamo, and may be able to constrain the bulk electrical conductivity.

## The Early Solar System Location of Psyche and M-type Asteroids from Dynamical Models

In this section, we discuss different scenarios for the formation location and early dynamical evolution of Psyche. We start by briefly describing constraints on Psyche and M-class asteroids that can help us evaluate dynamical scenarios.

The Main Belt is dominated in mass by a few large asteroids; for example, 1 Ceres has a third of the total mass (e.g., Vernazza et al. 2021). Thereafter, they follow a complicated size-frequency distribution that is often treated as a segmented or “wavy” power-law, that is, a cumulative number $N$ larger than $D$ going as $D^{-n}$, where $n$ varies with size. The size-distribution is relatively shallow for the half-dozen largest asteroids, and then becomes steeper (larger $n$, more asteroids of a given size) for asteroids smaller than around 300 km diameter. Then, for asteroids smaller than 100 km, smaller than Psyche’s diameter, the size-distribution transitions again to a shallower slope.

Bottke et al. (2005) used collisional evolution models to argue that most asteroids larger than about 100 km in diameter are original planetesimals, whereas smaller asteroids are increasingly the remnants of catastrophically disruptive collisions (e.g., Farinella et al. 1982). This fits with recent modeling work on planetesimal formation, where pebbles collect via streaming instabilities into spatially dense swarms that gravitationally collapse into planetesimals (e.g., Klahr and Schreiber 2021). The implication is that Psyche, being larger than 100 km in diameter, may represent a body that is potentially original and has possibly avoided catastrophic disruption. At face value this suggests that Psyche might not have originated from the disruption of a larger parent body; however see Sect. 3.

Psyche is classified as an M-type asteroid, an enigmatic taxonomic class with a red-sloped average disk-integrated spectrum that lacks evidence for significant absorption bands in visible to near-IR wavelengths (see review in Elkins-Tanton et al. 2020). In the DeMeo-Bus taxonomic system, these bodies are known as Xk (DeMeo et al. 2009). M-types have moderate albedos of 0.1 to 0.2, and this characteristic allows them to be distinguished from other classes of bodies that also have reddish slopes without absorption bands (e.g., dormant comets, which generally have albedos $<0.1$).

Finally, Psyche’s estimated bulk density of $3{,}780\pm340~\text{kg}\,\text{m}^{-3}$ (Elkins-Tanton et al. 2020) is comparable to three other M-types larger than 100 km: (21) Lutetia, (22) Kalliope, and possibly (216) Kleopatra. Vernazza et al. (2021) used high-angular-resolution imaging from VLT/SPHERE/ZIMPOL to estimate the 3D shapes for these bodies and calculated the densities of Psyche, Lutetia, Kalliope, and Kleopatra as $3{,}890\pm530$, $3{,}450\pm210$, $4{,}360\pm500$, and $3{,}450\pm410~\text{kg}\,\text{m}^{-3}$, respectively). Given these similarities, our working assumption here is that Psyche is a representative member of its taxonomic class.

To more closely examine M-type asteroids and compare them to other asteroids, we compiled the diameters and albedos of all asteroids in the AKARI and WISE datasets (Masiero et al. 2011; Alí-Lagoa et al. 2018). These data were then linked to (i) their taxonomic types as defined by DeMeo et al. (2009), Neese (2010) and DeMeo and Carry (2014) (with updates provided by F. DeMeo, personal communication), and (ii) their proper semimajor axis, proper eccentricity, and proper inclination values using the synthetic proper elements from the Asteroid Dynamics website AstDys (https://newton.spacedys.com/astdys/). With this combination, we identified all asteroids with diameter $D\geq40~\text{km}$ in the main belt; these bodies have not had their orbits meaningfully influenced by Yarkovsky thermal drift forces (e.g., Bottke et al. 2006a), this size range is observationally complete, and most of these bodies are likely to have formed in the first millions of years of the solar system, so we can use their present-day orbits to glean insights into their origins and early dynamical evolution.

From here, we combined large taxonomic classes with similar properties together and analyzed their dynamical distribution across the main belt, following the work of DeMeo et al. (2009). Our expectation is that dynamical excitation produced by early giant planet migration spread their eccentricities and inclinations values (except for a possible case we will discuss below) (Deienno et al. 2017, 2018), so here we concentrate on their semimajor axis distribution. In this manner, our work is similar to that performed by DeMeo and Carry (2014) (see also Gradie and Tedesco 1982), with diameter used as the primary discriminant rather than asteroid mass. The breakdown of our groups are as follows.

The two largest classes of S-complex bodies among our bodies are S- and L-type asteroids. The former are objects that have been linked to ordinary chondrites (e.g., Nakamura et al. 2011). The L-types have modestly different characteristics than S-types; a steep red spectrum shortward of 0.75 μm, a flat slope for wavelengths longer the 0.75 μm, and a moderate albedo (DeMeo et al. 2009, 2015). The S- and L-types have a similar semimajor axis distribution to one another, so for simplicity we put both sets together. Other S-complex asteroids have limited numbers of $D\geq40~\text{km}$ bodies, so they were excluded.

We divided asteroids resembling carbonaceous chondrites into several groups. The first group was D- and P-type asteroids, bodies whose spectroscopic signature often resembles dormant comets (e.g., DeMeo et al. 2009, 2015; Vokrouhlický et al. 2016). The second group was comprised of B- and C-type asteroids, bodies with spectra comparable to asteroids like Bennu, Ryugu, and Ceres (e.g., DeMeo et al. 2015; Hamilton et al. 2019; Kitazato et al. 2019). These taxonomic types make up the vast majority of all low albedo asteroids in the main belt (i.e., albedo $<12\%$).

Curiously, we found that about 90 main belt bodies with $D\geq40~\text{km}$ that have no taxonomic designation. These were predominately asteroids in the outer main belt with semimajor axis $a > 2.8~\text{au}$ and albedos $<8\%$. Rather than exclude these bodies, we created albedo probability distributions for the of D/P-types and C/B-types in the outer main belt and then assigned the unclassified bodies to each group according to their albedos. Specifically, we first placed the albedos of the known D/P-types and C/B-types from the outer main belt into bins with a range between 0 and 0.08, with the bin sizes being 0.01. Next, we added the number of bodies within a bin that were D/P type or C/B-types and then divided by the total, giving us a fraction for each within the bin. From there, we took the albedos of the unassigned bodies, found the bin they were in, and used random deviates to choose a taxonomic type. This method is approximate but reasonable, given that there should be no bias between the spectral identification of a D/P type vs a C/B-type in the outer main belt.

The third group was a small unusual class called K-types, whose spectra appear to be a match with CV and CO-type carbonaceous chondrites (e.g., Burbine et al. 2002a). An example K-type asteroid would be (221) Eos, the largest member of the Eos family, while an example CV chondrite would be the calcium-aluminum inclusion-rich (CAIs,the first solids of our solar system) meteorite Allende. K-types have moderate albedos and spectra that almost seem to be a combination of C- and S-types; for this reason, they were named for the letter halfway between C and S in the alphabet. Note that many K-type $D\geq40~\text{km}$ bodies are part of the Eos family, so all in the family but (221) Eos itself were excluded to avoid bias in our calculation.

**Fig. 3:**
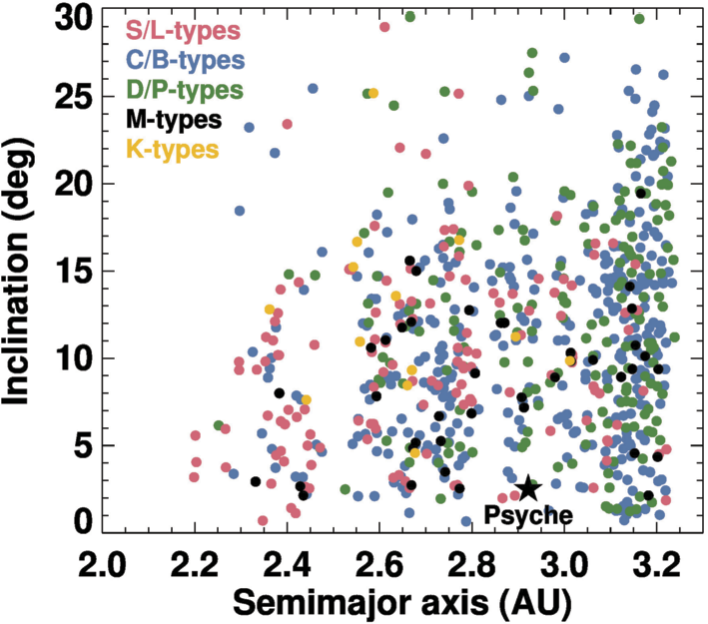
Orbital distribution of $D > 40~\text{km}$ asteroids in the main belt between 2.0–3.3 au that fall within the listed taxonomic groups: S/L-types (red dots), C/B-types (blue dots), D/P-types (green dots), M-types (black dots), and K-types (gold dots). All populations are dynamically excited, and there is no obvious evidence for major differences in their eccentricity and inclination distributions. The semimajor axis distribution of the classes are shown in Fig. 4

Finally, using the taxonomic catalog of M-types from our various catalogs, we found there were 41 M-types with $D\geq40~\text{km}$ in the main belt (Fig. 3). These bodies make up $\sim5\%$ of the $D\geq40~\text{km}$ population (out of 767 such objects between 2 and 3.3 au across all taxonomic groups). Many large M-types have semimajor axes $a$ between $\sim2.6$ and $\sim2.9~\text{au}$ (Scott et al. 2015). The remainder are located near $\sim2.4~\text{au}$ and $\sim3.1\text{--}3.2~\text{au}$. In general, M-types show a wide range of eccentricities ($e$) and inclinations ($i$), though in Scott et al. (2015), it was shown that for the eight M-types with $i < 5^{\circ}$ and $D\geq50~\text{km}$, three are at $a = 2.42~\text{au}$ and four more are at $a = 2.67~\text{au}$. We will return to this issue below.

**Fig. 4:**
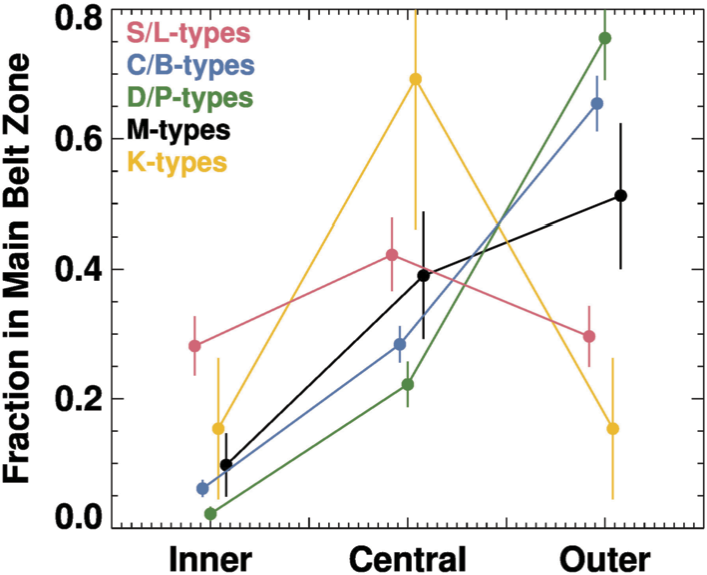
The fraction of $D > 40~\text{km}$ bodies in the main belt between 2.0–3.3 au with different semimajor axes and taxonomic classes. The listed taxonomic groups have the same colors as Fig. 3. We divided the inner, central, and outer main belt zones with divisions at 2.5 and 2.8 au, locations defined by the 3:1 and 5:2 mean motion resonances with Jupiter, respectively. For a given taxonomic group, error bars are the square root of the number of bodies within a zone divided by all objects in the group. The S/L-types have the largest fraction of inner main belt bodies, consistent with an origin that favors the inner solar system. The C/B and D/P have the largest fractions of bodies in the outer main belt, favoring an origin in the outer solar system. The M-types most closely resemble the C/B- and D/P-types distributions, suggesting that they may have originated in the outer solar system

To make approximate comparisons between M-types and other taxonomic groups, we have divided the asteroid belt into inner, central, and outer zones. We placed our divisions between these zones at 2.5 and 2.8 au, locations defined by the 3:1 and 5:2 mean motion resonances with Jupiter, respectively. Our results for $D\geq40~\text{km}$ asteroids are shown in Fig. 4, with the error bars calculated from the square root of the number of bodies in a bin.

Overall, we find that S/L-types have a relatively flat semimajor axis distribution across the three main belt zones, though they also have by far the largest fraction in the inner main belt. Conversely, the C/B-types and D/P-types have the strongest concentrations in the outer main belt. The M-types are closest to the distribution of the C/B-types, but there are differences as well, with the M-types having a greater fraction in the central main belt and a lower fraction in the outer main belt. Finally, the K-types seem relatively unique, with most of their bodies residing in the central main belt.

We caution that we have not renormalized the value for the fraction of the main belt depleted by the actions of giant planet migration (Nesvorny et al. 2017). This would increase the fractions in the inner main belt, which has experienced the most dynamical depletion, and would cause us to renormalize all of values. Tests suggest that the taxonomic distributions of different groups maintain their differences across the Inner, Central, and Outer main belt. For that reason, we decided not to modify the distributions using numerical modeling results, though it could be argued doing so provides a better picture of the main belt immediately after the end of giant planet migration.

To interpret Figs. 3 and 4, we need to consider what dynamical insights come from planet formation models. This subject does not make for easy distillation, so here we focus our attention on the results of numerical simulations that can reproduce many of the existing constraints (e.g., the masses and orbits of many solar system objects, including the terrestrial planets, asteroids in the main belt, and the Kuiper belt population).

Early in planet formation, when the solar nebula was present, non-carbonaceous planetesimals were growing in the inner solar system and carbonaceous planetesimals, with a range of rock and ice fractions, were forming in the outer solar system (e.g., Alexander et al. 2012; Kruijer et al. 2017; Kleine et al. 2020). The formation of Jupiter, or perhaps a pressure bump near where Jupiter formed, may have opened a gap in the solar nebula, preventing materials entrained in the gas from mixing between the inner and outer solar system (Kruijer et al. 2017; Brasser and Mojzsis 2020). This action is thought to explain the differences of isotopes in non-carbonaceous and carbonaceous meteorites (e.g., those of molybdenum Mo, ruthenium Ru, titanium Ti, and chromium Cr). It is now thought that non-carbonaceous chondrites formed in the inner solar system, while carbonaceous formed in the outer solar system, and that iron meteorites are associated with both groups. This implies that the metal-rich M-types could have presumably formed in either the inner or outer solar system.

Protoplanets and planetary embryos were also growing in these zones, though perhaps not in the asteroid belt region, via pebble accretion (where gas drag slows small objects enough that they are readily accreted by early-forming planetesimals) (e.g., Levison et al. 2015). The earliest bodies to accrete were most likely to differentiate, thanks to the heat given off by the decay of ^26^Al. Measurements suggest that the majority of our iron and stony-iron meteorite parent bodies formed before 300,000 years after CAI formation (Kruijer et al. 2014; Scherstén et al. 2006; see review in Scott 2020), and Vesta likely formed within 1.5 Ma of CAIs (Neumann et al. 2014). Psyche and other M-types are likely to have originated at this time, but their formation location is unknown.

In the terrestrial planet region, protoplanets grew and began to dynamically excite the planetesimal populations around them. This led to mergers, collisional evolution, and possibly hit-and-run events, where the crust and mantle of some large differentiated planetesimals would be stripped by impacts with similarly sized or somewhat larger bodies (e.g. Asphaug et al. 2006; Nesvorny et al. 2021). Given its expected bulk density of $\sim4{,}000~\text{kg}\,\text{m}^{-3}$, a single hit-and-run collision during the formation epoch could produce Psyche when a differentiated Vesta-sized progenitor collides with a still more massive (e.g., Ceres-sized) target. In this case, the Vesta-sized body escapes accretion (impacting too fast, or at too grazing an angle), losing much of its mantle in the process. Depending on the initial population that formed the planets, according to Asphaug (2010) the unaccreted population might include survivors of multiple hit-and-run collisions. This is also confirmed by several numerical studies of the late stage of terrestrial planet formation in the solar system (e.g., Chambers 2013; Emsenhhuber et al. 2020). Some of these survivors might consist primarily of mantle materials, and others may contain significant metallic core material. This scenario could apply to Psyche if it turns out to be primarily metallic (discussed in more detail below).

In the giant planet zone, similar events likely took place, and some planetary embryos were able to reach the size of several Earth masses via pebble accretion and embryo mergers (e.g., Kretke and Levison 2014). This allowed them to accrete a gaseous envelope from the solar nebula, and this core accretion scenario defines a leading hypothesis for how our system of giant planets was formed (e.g., Ormel and Klahr 2010; Lambrechts and Johansen 2012; Kretke and Levison 2014). If true, hit and run events in the outer solar system may have been common, as implied by simulations that show protoplanets and planetesimals frequently collide with one another in the same zones where the cores of the giant planets are being constructed (e.g., Levison et al. 2015; Raymond and Izidoro 2017a,b). It is highly likely that some bodies that experienced early collisional evolution in the giant planet zone made their way to the main belt.

Near the end time of the solar nebula, it is possible that a particular planet formation process took place that affected the orbits of many planetesimals. Specifically, proto-Jupiter may have grown large enough that its gravitational interactions with the solar nebula allowed it to migrate inward across the main belt zone. This event has been hypothesized to be the opening maneuver to what is now known as the “Grand Tack” (Walsh et al. 2011). Reaching $\sim1.5~\text{au}$, Jupiter may have then become trapped in a mutual 2:3 resonance with an inward-migrating proto-Saturn. At that point, the collective gravitational behavior of Jupiter, Saturn, and the solar nebula may have caused Jupiter and Saturn to move away from the Sun. The outward migration ended when sufficient gas dissipated from the solar nebula that Jupiter was no longer able to move via gravitational torques. Modeling work and certain constraints indicate that this could have occurred when Jupiter reached a semimajor axis of $\sim5.5\text{--}5.6~\text{au}$.

The Grand Tack and other dynamical models of planet formation and migration predict the gravitational scattering of small bodies in both the inner and outer solar system (e.g., Raymond and Izidoro 2017b). In these scenarios, S/L-type asteroids and other inner solar system bodies with characteristics representative of planetesimals from the Venus-Earth-Mars-Asteroid Belt zone were excited and then injected into the main asteroid belt. These bodies represent non-carbonaceous materials. Beyond Jupiter, C/B-type asteroids that were once denizens of the Jupiter-Saturn-Uranus-Neptune zone and beyond were captured into the main belt region. One of the strengths of the Grand Tack model is that it can plausibly reproduce the taxonomic distribution of large S/L- and C/B-type bodies found in the asteroid belt today (Walsh et al. 2011; see also Morbidelli et al. 2015).

The Grand Tack model was proposed as a means to explain the small size of Mars relative to Venus and Earth and the dynamical characteristics of the asteroid belt (Walsh et al. 2011), but it is not without its criticisms. The conditions required for, and plausibility of, the outward migration of Jupiter are still being explored (Raymond and Morbidelli 2014; Kanagawa et al. 2018). Additionally, the rapid formation of the terrestrial planets implied by the Grand Tack requires very efficient chemical equilibration during giant impacts, possibly inconsistent with fluid modeling studies (Zube et al. 2019).

Alternative models to the Grand Tack also exist. Of note, recent work has suggested that the terrestrial planets may have formed via pebble accretion (Levison et al. 2015; Johansen et al. 2021). The plausibility of this mechanism is in part controlled by an outstanding question of the ability of solids to migrate into the inner solar system after Jupiter has formed (Izidoro et al. 2021). Giant planet zone planetesimals may have been delivered to the main asteroid belt by a combination gas drag processes and scattering events produced by a growing Jupiter (e.g., Raymond and Izidoro 2017a,b; Kretke et al. 2017). There are also several other mechanisms that can potentially reproduce the small size of Mars (e.g., Levison et al. 2015; Clement et al. 2018; Johansen et al. 2021; Nesvorny et al. 2021). More work is still needed to fully explore the geochemical and dynamical implications of all of these planet formation models.

The other, independent, hypothesized dynamical event would have taken place after the solar nebula dispersed. At this time, the giant planets are thought to have been in a different configuration than the one seen today (Tsiganis et al. 2005). Gas accretion left the giant planets on nearly circular coplanar orbits between 5.5 and $\sim20~\text{au}$, with most or all of these worlds locked in mutual mean motion resonances with one another. The primordial Kuiper belt existed just beyond the original orbit of Neptune, with an estimated comet population of $\sim20$ Earth masses that stretched beyond 20 au. This configuration, however, is hypothesized to have eventually become unstable. As the primordial Kuiper belt lost planetesimals to the giant planet zone, it caused some giant planets to migrate far enough to either enter into or exit dynamical resonances with one another, and likely involved 5–6 giant planets, with 3 or 4 Neptune-sized bodies (e.g., Tsiganis et al. 2005; Nesvorný 2011; Nesvorný and Morbidelli 2012; Batygin et al. 2012; Vokrouhlický et al. 2016; see review in Nesvorny 2018). The culmination of these events led to the outermost giant planets migrating outward through the primordial comet disk in a violent exchange of orbital energy and angular momentum. This is thought to have led to a reorganization of the giant planets in what is now called a “giant planet instability”. This instability could have occurred with or without a Grand Tack earlier on (Deienno et al. 2018).

The most important factors for the origin of Psyche in the planetesimal evolution and giant planet migration scenarios discussed above are as follows. First, giant planet encounters with one another allowed a small fraction of bodies from the primordial Kuiper belt to be captured in the central and outer main asteroid belt, the Jupiter and Neptune Trojan populations, and the irregular satellite populations (e.g., Levison et al. 2009; Nesvorný et al. 2013; Vokrouhlický et al. 2016; see review by Nesvorny 2018). This mechanism explains why primitive D- and P-type asteroids are found in those locations. Second, asteroids in the main belt at the start of the giant planet instability were dynamically excited and depleted (Nesvorny et al. 2017; Deienno et al. 2018). Third, the giant planet instability may have occurred early, possibly as soon as the solar nebula dissipated (e.g., Nesvorny et al. 2018, 2021; de Sousa et al. 2020). Accordingly, migrating giant planets may play a key role in exciting the planetesimals and protoplanets located in the terrestrial planet region, even if the giant planets never migrated that far (Agnor and Lin 2012; Kaib and Chambers 2016; Clement et al. 2018; Nesvorny et al. 2021). Note that if the instability occurs too late, the induced excitation of the terrestrial planets would likely produce orbits for them unlike those seen today.

Thus, the population of the main asteroid belt, thought to contain bodies that originated from the inner and outer solar systems, could be explained as the net result of events that took place when the nebula existed (Grand Tack and/or gas drag processes) combined with events that took place after the solar nebula was gone (giant planet instability and migration). With our discussion of early dynamical processes in place, we can now consider how to use M-type orbital constraints to glean insights into the formation location and dynamical evolution of the M-types and Psyche.

### M-types from the Terrestrial Planet Region

One possible starting point for Psyche and the M-types is the terrestrial planet region. Dynamically, the primary ways such objects can get into the main belt zone early in solar system history is by scattering events from planetary embryos or gravitational interactions with Jupiter via the hypothesized Grand Tack (Bottke et al. 2006b; Walsh et al. 2011; Raymond and Izidoro 2017a; Kretke et al. 2017). Regardless of which mechanism is used here, these interactions predominantly place terrestrial planet zone planetesimals into the inner and central main belt regions, the orbital location where most large S/L-type asteroids are found today. Our expectation is that if the M-types formed in the same locale as the S/L-types, they should have been delivered to the main belt by the same mechanisms and therefore should have a similar distribution to the S/L-types. The problem is that few M-types exist in the inner main belt, a mismatch with these model predictions (Figs. 3 and 4).

A possible way out was suggested by Bottke and Asphaug (2013) (see also Scott et al. 2015), who showed that some large M-types have semimajor axis locations consistent with “fossil resonances” of Jupiter that existed when Jupiter was located at $\sim5.55~\text{au}$, its putative location at the end of the Grand Tack. Prior to the giant planets taking on their present-day orbits, Jupiter was likely on a circular orbit, and many of its resonances within the main belt would have produced trapping rather than diffusive behavior. Numerical simulations showed that low eccentricity objects encountering such a resonance could enter the main belt zone. If Jupiter later migrated to its current orbit of a $\sim5.2~\text{au}$ via a giant planet instability, these objects would become permanently trapped in the main belt.

This scenario could potentially explain the capture of low inclination (i.e., $<7^{\circ}$) M-types at the 7:2 and 3:1 mean motion resonances with Jupiter. For Jupiter at $a\sim5.55~\text{au}$, these resonances would be at 2.4 and 2.67 au. The problem, however, is the timescale. Simulations of this behavior indicate that putative M-types could take many tens to hundreds of millions of years to enter fossil resonances (Bottke and Asphaug 2013; Raymond and Izidoro 2017a). This is permissible if the giant planet instability occurs tens to hundreds of millions of years after the solar nebula is gone. If it occurs millions of years after the dispersion of the nebula, however, there is not enough time to get a substantial population of M-types into the main belt zone through these “dynamical doors”.

For these reasons, we infer that the M-types are unlikely to be inner solar system bodies, in which case Psyche is unlikely to be made of non-carbonaceous materials.

### M-types from the Asteroid Belt

It is possible that the M-types were not implanted but instead are indigenous to the central main belt. If true, the putative population could not have been dynamically affected in a profound way by the putative Grand Tack, which would have scattered M-types throughout the main belt. Any model of M-type formation and evolution would also need to explain why M-types are distributed in semimajor axis in a manner similar to C/B-types and D/P-types (Figs. 3 and 4).

Another potential problem with forming M-types in the main belt would be to explain how many of them ended up in the fossil resonances of Jupiter (Scott et al. 2015). We suspect that a population that originated in the main belt would have difficulty matching the dynamical constraints of the low inclination M-types, but this would need to be considered within a model of main belt evolution that can meet other dynamical constraints. On the other hand, it is also possible that the fossil resonance argument is weaker than suggested.

At this time, we find no strong dynamical evidence in support of the idea that M-types formed in the main belt zone. More planetesimal formation and dynamical evolution work is needed to determine whether a plausible solution exists for this hypothesis.

### M-types from the Primordial Kuiper Belt

It is also possible that the M-types originated beyond Jupiter and are in fact carbonaceous in nature. In this circumstance, we need to examine dynamical processes that can capture putative M-types in the central and outer main belt but also explain their limited numbers in the inner main belt relative to other populations.

One possibility is that the M-types were originally part of the primordial Kuiper belt, a population that was once thought to have totaled $\sim20$ Earth masses. Simulations indicate that approximately 99.9% of the population was scattered onto giant planet-crossing orbits when the giant planet instability caused Uranus and Neptune to migrate through the disk. A tiny fraction of this material would have been captured in stable small body reservoirs in the inner solar system, such as the central and outer main belt and the Hilda and Jupiter Trojan populations (e.g., Vokrouhlický et al. 2016). P-types are of particular interest because their spectral signatures in the visible to short-wave near-IR are similar to that of M-types in that neither has strong absorption bands (see review by DeMeo et al. 2015). The main discriminant between the two taxonomic types is not spectra but albedo, with P-types generally having albedos much less than 10% percent and M-types having albedos that are 10–20%.

This leads to the prediction that if substantial numbers of M-types were coming from the primordial Kuiper belt, we would expect some M-types to have also been captured in the Hilda and Trojan asteroid populations, given the substantial number of large M-types found in the asteroid belt. Accordingly, the question is whether M-types could remain relatively “hidden” among a large P-type population such as the Trojans. At least so far, it appears the answer to this question is no. Observational evidence in the form of extensive Trojan observations indicate they have uniformly low albedos of around 0.05 (e.g., Emery et al. 2015).

We conclude that M-types are unlikely to be from the primordial Kuiper belt, unless their true nature can be easily hidden and that same camouflaging process works much less efficiently in the main belt, where we see M-types, than in the Hilda and Trojan populations.

### M-types from the Giant Planet Zone

The evolution of planetesimals formed in the giant planet zone may provide a more plausible means to explain the M-types by implantation. An examination of the dynamical results from Walsh et al. (2011) shows that asteroids originally located between the giant planets can be dynamically captured within the main belt. This model can explain the orbital distribution of the C/B-types, and we find it interesting that the M-types have a similar semimajor axis distribution to this class (Figs. 3 and 4). That could suggest a common origin location. If there is no Grand Tack, the implantation would instead take place by gas drag processes combined with scattering events from a growing Jupiter (e.g., Raymond and Izidoro 2017a, 2017b; Kretke et al. 2017).

The capture location of giant planet zone bodies depends on the starting location within the giant planet zone. Numerical simulations of the Grand Tack or giant planet formation combined with gas drag processes suggest that giant planet zone planetesimals that are closer to Jupiter show a greater likelihood of being captured in the inner and central main belt than those that are further away from Jupiter (Walsh et al. 2011; Raymond and Izidoro 2017a, 2017b; Kretke et al. 2017). Based on this, we postulate that the M-types and Psyche were came from a zone that was closer to Jupiter than in a zone that produced most C/B-types. It has already been argued that certain carbonaceous meteorite types may have formed near Jupiter, such as the water-poor but CAI-rich CV chondrites (e.g., Desch et al. 2018). Our hypothesized scenario would potentially explain why M-types have a greater fraction of central main belt bodies and a lower fraction of outer main belt bodies than C/B-types (Figs. 3 and 4). We note that this idea is broadly consistent with existing numerical simulations but has yet to be tested by any specific simulation.

Another issue to investigate is whether some fraction of the M-types from the giant planet zone could find their way into the main belt via Jupiter’s fossil resonances. For example, in the Grand Tack, M-types would get into the main belt as Jupiter migrates outward, but the capture events would happen near the migration endgame. Here the solar nebula would be nearly dissipated, and Jupiter would be grinding to a halt near its final Grand Tack location of $\sim5.55~\text{au}$. This would leave M-types adjacent to the main belt to find those dynamical doors that existed at that time, namely the fossil resonances. Perhaps a comparable mechanism would take place with gras drag implantation. Our cautionary note is that there may not have been much time for them to get into the main belt if the giant planet instability occurred shortly after the end of the solar nebula.

Finally, we find it intriguing that the K-types are concentrated in the central main belt. K-types are thought to produce CV and CO chondrites (Burbine et al. 2002a), unusual carbonaceous chondrites that have a lower abundance of volatiles compared to CM or CI chondrites. Their CAI abundances are also quite high relative to other classes. It can be argued that CAIs originally formed near the Sun and were spread out through the solar nebula prior to the formation of Jupiter’s core (e.g., Desch et al. 2018). Once Jupiter became large enough to cut off nebula communication between the inner and outer solar system, CAIs in the outer solar system would tend to evolve inward toward the Sun via gas drag. The net result would be higher concentrations of CAIs in a zone to the anti-sunward Jupiter than in other zones, and planetesimals forming there would be rich in CAIs (Desch et al. 2018). At that point, dynamical mechanisms akin to those discussed above would place a small fraction of these planetesimals into the main belt.

At this point, we engage in further speculation based on the limited information that exists, though the reader should be aware that the threads of evidence are thin. The results in Figs. 3 and 4 show that K-types have a different taxonomic distribution than any other group that is shown, while the M-types appear to have a distribution closer to that of the C/B group. If these trends are not dominated by small number statistics, the distribution differences could be telling us about their source regions. If we further assume that K-types are related to CV/CO chondrites, and they formed near the Jupiter zone, that would potentially rule out the possibility that the M-types formed in that zone, unless they formed at a different time (and that influenced how they get into the main belt). Finally, if we assume every asteroid group had to form in a distinct zone within the giant planet region, it forces us to put the M-types in a different place than the C/B-types and the D/P-types. We argued above that the D/P-types probably came from the primordial Kuiper belt, which leaves us with the M-types and C/B-types. If we were forced to choose an order for them, we would look to the semimajor axis distribution of the M-types, which potentially split the difference between the K-types and the C/B-types (Fig. 4). If true, this would indicate that M-types originally resided in the outer solar system between the K-types, which formed fairly close to Jupiter, and the C/B-types, which formed further away from Jupiter.

To melt and form a core as a planetesimal, Psyche would have had to accrete within $\sim1.7~\text{Myr}$ after CAIs if it contained the canonical concentration of ^26^Al. If Psyche is part of a differentiated planetesimal, and it originated in the outer solar system, it would have had to either accrete earlier than the 3 to 4 Myr after CAIs assumed for most carbonaceous chondrite parent bodies, or it had to have originated with more ^26^Al. This scenario only holds if Psyche is a part of the core of a planetesimal. If it was part of a larger body, or a droplet from a much larger collision, then the timing constraints are removed.

To match existing Psyche observations, a possible origin in the volatile- and carbon-rich outer solar system would have to be followed by crustal and mantle stripping, to accord with the relatively high bulk density of Psyche. This would include loss of exterior volatiles and any ice-shell, even in low-velocity (shock-free) mantle stripping that does not cause much heating, simply by virtue of being in the exterior and lowest gravitational potential. Further, the heating of a differentiating planetesimal liberates fluids long before metal-silicate separation, and these fluids are likely to be lost to space (Young 2001; Young et al. 2003; Fu and Elkins-Tanton 2014).

However, depending on the size of the parent body and its own thermal evolution, hydration may exist throughout the body. For example, Titan is proposed to be dominated by a mantle composed of hydrated silicates (e.g., Fortes 2012) and may even possess a hydrated core. In such a case water would be difficult or impossible to remove even in an event that removed nearly all the mantle, which is not likely in any case. In this case, these hydrated phases, stable in the original hydrostatic parent body, might not be stable near the mantle-stripped Psyche’s surface, so volatiles might be expected to be present, or evidence for volatiles driven off to space with evidence for sublimation degradation and associated mass wasting.

There is much to investigate here, but we speculate that it may be possible to make a case that the M-types formed in the giant planet zone and were implanted in the central and outer main belt by the Grand Tack or by gas drag processes related to the formation of the giant planets. Their evolution in the giant planet zone could also open the door for hit and run-type encounters to be involved with their evolution, given that the formation of the cores of the giant planets led to many such events (e.g., Levison et al. 2015).

## The Fiducial Origin Scenario: A Differentiated Parent Body for Psyche

Psyche’s apparent composition of metal plus lower-density material, and the inferred paucity of FeO in that lower-density material, place constraints on its possible formation. These formation processes may also in some cases be aligned, or misaligned, with the possibility that Psyche originated in the outer solar system, as described above.

The first and simplest way to accumulate a high fraction of metal is through melting and differentiation of a body into a metal core and a silicate mantle.

### The Process of Core Formation in Planetesimals

Core formation in planetesimals likely begins at the onset of partial melting under high-temperature, low-pressure and relatively dry conditions. Melting in planetesimals must be almost exclusively driven by the heat of radiogenic ^26^Al (Fish et al. 1960; Huss et al. 2006; LaTourrette and Wasserburg 1998; Lee et al. 1976; Urey 1955). This is in sharp contrast to well-known adiabatic melting regimes on Earth, which occur at depth in the upper mantle at pressures up to 24 GPa (240 kbars) or above, or under wet crustal conditions where melting can begin at $\sim800~^{\circ}\text{C}$. Even the centers of the largest differentiated asteroids (e.g., Vesta) reach only pressures of $\sim2~\text{kbars}$. The first silicate partial melts on asteroids (generally picritic, with SiO_2_ below 52 wt%, and MgO up to 30 wt%) first occur when temperatures reach $\sim1050~^{\circ}\text{C}$. However, the very first melts of a chondritic precursor are not basaltic, but Fe,Ni-FeS melts that form at $\sim950~^{\circ}\text{C}$ and contain $\sim85$ wt.% FeS (Kullerud 1963).

Numerous experimental studies (e.g., Gaetani and Grove 1999; Miller et al. 2016) demonstrate that because of their surface tensions these melts will not readily migrate under static conditions. Yet, melt migration did occur at low degrees of partial melting in asteroids. The preponderance of FeS (troilite) within these early formed melts and its relatively low abundance in chondritic meteorites ($\sim5$ wt.%) makes tracing S and/or troilite abundances a particularly powerful tool for understanding early partial melting in meteorites collectively dubbed primitive achondrites.

This group, including acapulcoites-lodranites, winonaites/IAB irons, brachinites, and ureilites, exhibit broadly chondritic compositions and mineralogies with evidence of partial melting (McCoy et al. 1996, 1997a,b; Mittlefehldt et al. 1996). Although micrometer- to centimeter-sized metal-sulfide veins are present in acapulcoites, significant sulfur depletions are only common in the lodranites, where silicate melting opened pathways for melt migration. Evidence for silicate melting and melt migration is found in plagioclase abundances. Basaltic partial melt contains $\sim55$ wt.% plagioclase stiochiometrically (Morse 1980) and chondritic meteorites contain $\sim10$ wt.% plagioclase. Consequently, plagioclase (or its constituent elements, most notably Al, Ca, Na, and K) is rapidly depleted in residues during partial melting. Plagioclase content is therefore a tracer for early silicate melting.

Melt migration at low degrees of partial melting may not have been a static process, but a dynamic process driven by overpressure resulting from melt expansion and/or release of volatiles, notably CO and S (McCoy et al. 1997b). The fate of these Fe,Ni-FeS and basaltic partial melts is uncertain, but two tantalizing possibilities exist in the absence of higher degrees of partial melting. Keil and Wilson (1993) suggest that on bodies only a few tens of kilometers in size, Fe,Ni-FeS melts could be buoyant in the presence of released volatiles, driven to the surface, and erupted into space at velocities exceeding the escape velocity of the body, possibly explaining the S-depletion in some iron meteorite parent bodies. On larger bodies like Psyche, these Fe,Ni-FeS melts could migrate to the center of the body, forming essentially a proto-core much smaller and richer in S than formed during later migration of metal during high degrees of partial melting more typically associated with core formation. These small S-rich proto cores may be sampled by meteorites like some of the silicate-bearing IAB irons.

Low-degree melting, however, may not have been a dominant process in planetesimal formation. Isotopic evidence from iron meteorites shows that their parent bodies accreted within 100,000 to 300,000 years after formation of calcium-aluminum inclusions (Kruijer et al. 2014; Scherstén et al. 2006; see review in Scott 2020). Results of pebble accretion models show that, given planetesimals of tens to hundreds of kilometers radius and the continued existence of a gas disk, gas giants the size of Jupiter can accrete in the outer solar system, in only an additional 100,000 years (Lambrechts and Johansen 2012; Ormel and Klahr 2010; Pollack et al. 1996). Thermal and compositional models of planetesimals show that if they formed within $\sim1.7~\text{Ma}$ after calcium-aluminum inclusions the heat of ^26^Al would be sufficient to melt them completely (Elkins-Tanton et al. 2011; Sahijpal et al. 2007; Šråmek et al. 2012).

Once melting has progressed well past the eutectic, the relative densities of the materials determine whether differentiation occurs. In rapidly-accreted planetesimals that melt in the first $\sim1.7~\text{Ma}$ of the solar system, formation of metal cores was rapid and efficient. Thus a generation of “Ur-cores,” original, first-generation cores, would form in planetesimals, and then be mixed and reformed in all subsequent accretionary collisions. These subsequent collisions occurred at a rapid pace in the first few million years of the solar system, and then more slowly as populations waned and the oligarchic stage of planetary formation commenced (Chambers 2008). The gas disk had likely locally dispersed between $>1.22$ and $<3.94~\text{Ma}$ after CAI-formation in the noncarbonaceous reservoir, and between $>2.51$ and $<4.89~\text{Ma}$ in the carbonaceous reservoir (95% confidence limits) (Weiss and Bottke 2021).

Especially in the outer solar system, heating is reduced not only by the possibly longer time scale of accretion and diminution of ^26^Al, but also by the water content of the accreting materials. Castillo-Rogez and Young (2016), however, demonstrated that even with 50% water ice, if the planetesimal formed prior to 1 Myr after CAIs it could still reach the silicate solidus and differentiate into a metal core and silicate mantle. Thus, differentiation in planetesimals likely occurred in both the inner and outer solar systems, and this fiducial model for Psyche can be considered in either case.

### Planetesimal Processes that can Produce a Low-FeO Non-metallic Phase in Psyche

The bulk compositions of the non-metal fractions of almost all meteorites are too FeO-rich to satisfy Psyche’s spectral constraints (Dibb 2021). Thus, in this section, we present processes that can produce a low-FeO, low-density second phase that will satisfy both the spectral and density constraints of Psyche.

The melting of the parent planetesimal to allow metal core differentiation is a starting place. Fractional solidification in the molten silicate portion of a hot young planetesimal produces first high-MgO mafic minerals, gradually transitioning to more and more iron-rich mineral compositions as liquids evolve. In larger bodies, settling timescales can be significantly less than solidification timescales (Suckale et al. 2012), and thus a radial structure with high MgO at the bottom and high FeO at the top is produced. These cumulate piles are gravitationally unstable, with both thermal and compositional parameters increasing density at the top of the pile, and will overturn in the solid state (e.g. Ringwood and Kesson 1976; Spera 1992; Solomatov 2000).

In larger bodies, if the outer mantle can be removed before overturn, the body will be left with an iron-nickel core, and only the lower-mantle high-MgO, low-FeO silicates of the initial solidification remaining. Mechanisms for removing that upper mantle are complex and not well understood. Removal without mixing is a challenge, as is understanding the effects of depressurization and oxidation.

In the process of fractional solidification at the low pressures of planetesimal mantles, almost all chondritic compositions would first crystallize olivine (summary in Elkins-Tanton 2017 and see Okada et al. 1988), with the enticing exception of enstatite chondrites, which would first crystallize orthopyroxene (McCoy et al. 1999). This observation is enticing because Hardersen et al. (2005) suggested based on spectra that the non-metal phase on Psyche is orthopyroxene.

As soon as crystallization extends throughout the molten mantle, separation of crystals through floating or settling becomes difficult due to crystal interference and high viscosity (Elkins-Tanton 2017). Unlike the high gravity and often slower cooling conditions of larger planets, the solidus and liquidus of a planetesimal’s silicate mantle have very little change in temperature between the core-mantle boundary and the surface. Therefore only a small amount of additional cooling past the point where the adiabat crosses the liquidus will move the adiabat entirely between the solidus and liquidus, and crystallization will commence throughout the vertical column of the planetesimal mantle. Over the $\sim0.5~\text{kbar}$ mantle pressure range of a planetesimal $\sim200~\text{km}$ in radius, the solidus will change by only about $10~^{\circ}\text{C}$ and the adiabat by only $\sim2~^{\circ}\text{C}$ (Elkins-Tanton 2017). A body this size would likely be the smallest possible parent body for Psyche, if Psyche is mainly a differentiated core.

Settling of crystals during solidification on planetesimal or planetary scales remains poorly understood, but geochemical and mineralogical evidence for settling (Morse 1982) and floating in the lunar magma ocean is widely accepted (e.g., Smith et al. 1970; Warren 1985; Wood et al. 1970). The difficult relevant question here is over what solidification interval settling can occur successfully on bodies with low gravity, that is, how thick a layer of low-iron early-forming cumulates can coat the core of Psyche’s parent, ready to be incorporated into the Psyche of today while excluding the higher-iron, later-forming cumulates?

**Fig. 5:**
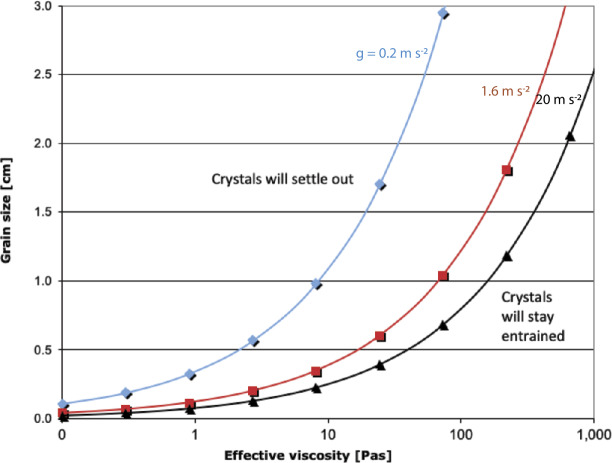
Only the largest early crystals forming in a planetesimal magma ocean will settle, and only until the effective viscosity rises past a certain point. In the low gravity of a planetesimal, crystals are likely to settle only while the magma ocean remains at viscosities of picritic melts, and before a significant crystal fraction grows. Thus only the earliest-forming mineral (almost always olivine, but for enstatite chondrites, orthopyroxene) will settle to the core-mantle boundary

Solomatov and Stevenson (1993) derive an expression for the size of particles that will settle during solidification (Eq. (12)):

$$ D= \biggl( \frac{10}{\Delta \rho g} \biggr) \biggl( \frac{\eta \alpha gF}{C_{p}} \biggr)^{0.5}, $$

where $D$ is grain size, $\Delta \rho $ is the density difference between the liquid and crystal, here taken as $100~\text{kg}\,\text{m}^{-3}$; $g$ is the gravitational acceleration of the body, here values for Vesta, the Moon, and Mars are used; $\eta $ is effective viscosity; $F$ is heat flux from the cooling body, here taken as $10^{4}~\text{W}\,\text{m}^{-2}$; $\alpha $ is the thermal expansivity, here $3 \times 10^{-5}~\text{K}^{-1}$; and $C_{p}$ is heat capacity, taken as $1{,}256.1~\text{J}\,\text{K}^{-1}\,\text{kg}^{-1}$ (Fig. 5). To settle on a small body like Vesta, crystals must grow rapidly to several millimeters while the effective viscosity of the liquid remains below 1 Pa s or so, which is consistent with an ultramafic or mafic largely crystal-free magma (Reese and Solomatov 2006). On small bodies, a critical crystal fraction forms rapidly because of the similar slopes of the adiabat and solidus, and settling of an individual phase would be impossible because of interference and networking among the solids.

Only the earliest crystals to form, therefore, have the ability to settle to the core-mantle boundary. These would be olivine in most cases, and pyroxene in the case of an enstatite chondrite bulk composition. If Psyche’s low-density phase is in fact low-iron enstatite (Hardersen et al. 2005) but its bulk composition was more oxidized than enstatite chondrites, its first-crystallizing olivine must have been reduced to pyroxene, possibly through extended contact with metal.

Several processes could have produced Psyche’s metal + low-density phase composition. The main goal of the three scenarios listed below is to produce a low-FeO, low-density second phase (or phase assemblage), satisfying the spectral observations of Psyche, that can be mixed with iron and nickel metal to produce the observed density of Psyche.

1. A Vesta-sized parent body is the smallest body consistent with Psyche’s size and density. In this scenario, a fully molten Vesta-sized parent body settles low-iron olivine or orthopyroxene in the earliest period of magma ocean solidification. Subsequently, the overlying cumulate mantle is removed by a series of impacts. A challenge to this scenario is the likely thinness of the olivine- or orthopyroxene-only layer at the bottom of the mantle due to the barriers to crystal settling. Only a specific series of collisions could strip only the outer mantle and leave the low-FeO silicates on the resulting Psyche.
2. A similar but possibly more plausible scenario is that Psyche’s parent body was a larger body, perhaps even Moon-sized, and then suffered one or more hypervelocity collisions. This larger body allows a thicker layer of iron-poor silicates to settle to its core-mantle boundary during solidification, and subsequent impacts need only retain a portion of the core and lowest mantle to become Psyche.
3. Psyche’s parent body went through the differentiation process and stripping processes described above, but its bulk composition was so reduced to begin with that the non-metallic portion was already low in FeO. Then, stripping via impacts is only required to remove sufficient silicates to match the compositional requirements. Though a simpler process, we have no examples of a meteoritic bulk composition with a silicate fraction so low in FeO.

### Impact Scenarios that Could form Psyche by Stripping Mantle Material

A fully differentiated planetesimal consisting of a silicate mantle and a metallic core, with the bulk composition of almost any chondritic meteorite, would have too low a bulk density to match Psyche (with addition of expected porosity, all chondrites are less dense than the asteroid appears to be with current data), and thus would need both the removal of silicate and the exposure of metal on the surface to match Psyche observables. Thus, we consider three scenarios: The gradual erosion of the parent body by relatively small impacts, the catastrophic disruption of the parent body by one giant impact, and the stripping of the body by hit-and-run impacts.

Each scenario has testable implications. Erosion by impacts, for example, would probably start with a solidified planetesimal. Imagine a solidified $\sim500$-kilometer diameter differentiated planetesimal being eroded over time by hundreds of impacts comparable in magnitude to the impact that formed the large Rheasilvia Basin on Vesta (e.g., Turrini 2014). The eroded body would always retain some remnant of the mantle, but would become increasingly dominated by a well-mixed megaregolith with grains consisting of ultramafic silicates, iron-silicate breccias, and metallic iron. In this scenario the exterior of Psyche would be this highly-processed mixture of material, including parts of the impactors. For this to happen to Psyche and not other major Main Belt asteroids like Vesta and Ceres, would perhaps require it to happen before its implantation in the Main Belt.

For larger bodies, the thermodynamics becomes significantly more complex, because the parent body may well be molten, given its much larger size, with pressures of $\sim10\text{--}100$ kilobars or greater. Apart from any other reactions, unloading makes available considerable enthalpy (Asphaug et al. 2011). Also, giant impact and planetary differentiation models suggest that unloading may chemically reduce deep-mantle materials (Cambioni et al. 2021).

One prediction would be the efficient degassing of any escaping deep-mantle clump ejected by a disruptive impact to form Psyche; pressure release makes available significant enthalpy. The available enthalpy has implications not only for super-catastrophic giant impacts, but for smaller disruptions as well, where a change in pressure can lead to significant disequilibrium and the expansion and release of dissolved volatiles as well as droplet production.

In general, if Psyche is a largely-metallic core of a parent body, and is itself mostly iron, then by whatever mechanism it lost its mantle, it began as an approximately Vesta-sized body or larger. But to consider mantle removal from a Vesta-sized parent body by only one or two impacts rather than by erosion, one really has to think of two parent bodies. At the $\sim5~\text{km/s}$ velocities typical in the modern Main Belt, the projectile itself would have to be as large as Psyche (Asphaug 2010). The prediction in that case would be a globally-shocked asteroid leading to the exposure of deep mantle rock and metal.

If Psyche’s progenitor was larger, e.g. a Moon-sized body, then its mantle removal by a hypervelocity collision would have to be proportionally even more intense; the projectile itself would have to be Moon- to Mars-sized, and Psyche would be a small remnant of one of the colossal “late stage” events of planet formation.

In the hit-and-run scenario (Asphaug 2017), Psyche is the unaccreted projectile (the “runner”) or remnant thereof, with little compositional connection, other than coincident location, to the target. They runner and target are bodies in an accreting swarm. Because hit-and-run impacts happen during the process of accretion, the progenitor may have been molten, within the million-year timescale of ^26^Al decay. The target that the proto-Psyche ran into (assuming proto-Psyche was originally Vesta-sized) would have been a larger planetesimal, perhaps twice as big, feeding on the more numerous smaller ones. Quite unlike ballistic disruption, mantle stripping by hit-and-run is at low velocity. During planetesimal accretion the impact velocities would be within the range of a few times the escape velocity of the larger bodies, less than 1 km/s for a Ceres-sized population, lower than the sound speed in geologic materials. The removal of a $>100$-km of mantle rock over the course of an hour (the gravitational encounter timescale) would have metamorphic effects on petrology, especially if the excavated material was volatile-rich or hydrated.

We think that a hit and run event is the most likely process for creating Psyche, but only if planetesimals grew hierarchically from smaller planetesimals. If they formed effectively instantaneously via pebble accretion, all the mechanisms are probable.

One question in understanding Psyche’s progenitor is the absence of a collisional family. In scenarios where Psyche’s parent body was disrupted or stripped before Psyche was implanted into the asteroid belt, its collisional family would be long ago lost by dynamical disruption, integration into growing planets, or loss into the Sun. Thus, the lack of a dynamical family is unsurprising, but argues against scenarios where it is disrupted in situ.

Finally, a note on processes that might happen after stripping. During solidification of metal, no matter the size of the parent, a segregated immiscible FeS liquid may be able to erupt either to the surface of the core (Abrahams and Nimmo 2019), or possibly through some remnant mantle and onto a silicate surface (Johnson et al. 2019). The Psyche spacecraft has been designed since 2012 to be able to measure sulfur on the surface of Psyche, in the hopes of detecting this possible ferromagmatism. Ferromagmatism would be a strong indicator that Psyche is the remnants of a core, that is, a mass of Fe-Ni-S metal that was once molten. But the ferromagmatism hypothesis itself does not form a complete model to explain Psyche’s spectral and density characteristics. In the Johnson et al. (2019) model, the mantle still must have a low-FeO characteristic and thus the body must have followed one of the stripping, erosion, or hit-and-run scenarios described above. And in the stripped core model of Abrahams and Nimmo (2019), Psyche would need extreme porosity or a second low-density phase to explain its bulk density, thus also requiring one of the above scenarios.

## Alternative Origin Scenarios

### Metal-Rich Chondritic Meteorites: A Possible Analog if Psyche Is not a Core

We consider whether some metal-rich chondrites might serve as appropriate analogs. In particular, we focus on groups of carbonaceous chondrites that share common properties, including the CR, CH, CB, and G chondrites, along with several ungrouped or intermediate meteorites. Weisberg et al. (1995) suggested grouping CR, CH and CB chondrites as the CR clan, indicating petrogenetic kinship. We hesitate to use this nomenclature both because of the inclusion in this discussion of the isotopically-distinct G chondrites and confusion that clan designation could imply a common parent body.

The most distinctive common features of these meteorites are abundant metal, and silicates that are poor in FeO. In this respect, these meteorites are tantalizing as possibilities to match the density and inferred silicate to metal ratio and the presence of low-FeO silicates (Dibb 2021; Hardersen et al. 2005) of Psyche. Metal abundances in these meteorites range from $\sim7$ vol.% in CR chondrites to more than 60 vol.% in CB chondrites (Weisberg et al. 1995). At the lower end of this metal range, the CR chondrites contain no more metal than the enstatite chondrites (Keil 1968). The average density of fragments of Bencubbin, the meteorite after which CB chondrites were named, is $5{,}300~\text{kg}\,\text{m}^{-3}$ (Simpson and Murray 1932). Olivine and pyroxene compositions range widely in some of these groups, with CR chondrite olivine ranging up to Fa50 (where $\text{Fa}=\text{molar}(\text{Fe}/(\text{Fe}+\text{Mg}))$, but all show strong modes in the range of Fa1 to Fa4, consistent with the low-FeO content of mafic silicates inferred for Psyche from a weak $\sim0.9$ micrometer absorption feature. The Psyche mission payload should be able to constrain the silicate-metal ratio from bulk density and composition, and the mafic silicate FeO relative areal abundance from multi-spectral imaging (Bell et al. this journal).

While these meteorites share a number of common properties, they differ in important ways indicative of formation mechanisms and locations. Notable among these differences are the size, texture, isotopic composition and ages of the silicate spheres (Weisberg et al. 1995). While none of these features can be determined from orbit, they are indicative of a complex set of origins. At the extremes, CR silicate spheres are mm-sized chondrules formed in the nebula, whereas cm-sized CBa silicate spheres formed $\sim1\text{--}2~\text{Ma}$ after nebular dissipation and appear to be the products of rapid cooling of condensates from an impact plume formed during planetesimal collision (Bollard et al. 2015; Krot et al. 2005). CH chondrites show a mixture of these two formation mechanisms (Krot et al. 2010) and the intermediate Isheyevo meteorite illustrates remarkable sedimentary textures interpreted as deposition from that plume (Garvie et al. 2017). The scale of impact plume deposition, and the possibility of resulting metal enrichment remains an open question.

The inclusion of the G chondrites (Weisberg et al. 2015), which are petrologically similar to the CBa chondrites, suggests that the process that formed these meteorites was widespread in the early solar system. The CR, CH and CB chondrites have isotopic compositions similar to other carbonaceous chondrites, presumably having formed in a common isotopic reservoir in the outer solar system (Warren 2011). In contrast, the eponymous G chondrite GRO 95551 has oxygen and chromium isotopic compositions suggestive of formation with the non-carbonaceous chondrites in the inner Solar System. Thus, impacts that produced meteorites with the cm-sized, quickly cooled silicate clasts and abundant metal typical of these meteorites occurred throughout the Solar System.

Chondrules in CB chondrites formed at $\sim3.8\pm1~\text{Ma}$ and $4.81\pm0.26~\text{Ma}$ after the nominal CAI-formation time (Amelin et al. 2010; Connelly et al. 2012). Large collisions between protoplanets would perhaps be expected within the giant planet zone at late nebula times when large bodies were more common. We speculate that these putative collisions could explain the existence of CH and CB chondrules, which presumably would form into M-types by planetesimal formation processes prior to injection in the asteroid belt. It could also explain the M-types as the byproduct of large and/or possibly hit-and-run collisions that could produce stony iron and iron meteorites.

If CR chondrites are primordial and formed beyond the snow line (Warren 2011), where CB chondrites formed by condensation of a high-temperature impact plume (Krot et al. 2005), it is not surprising that they also show differences in degree of aqueous alteration (Weisberg et al. 1995). CR chondrites contain ubiquitous aqueous alteration of the matrix (they could have up to 5 wt% water-equivalent H, per Schrader et al. 2014 and Alexander 2019, for example), while CH and CB chondrites contain only rare aqueously altered clasts, possibly remnants of the pre-impact assemblage (Greshake et al. 2002). This distinction, indicative of the history of the groups, should manifest in differences in hydrogen abundance determined with GRNS. If metal-rich CB chondrites formed by localized impact plume production on an otherwise aqueously altered body poorer in metal, orbital chemical measurements should detect the difference between these lithologies. This scenario is particularly intriguing given the detection of a weak $\sim3~\upmu \text{m}$ spectral feature indicative of water of hydration and rotational heterogeneity (Takir et al. 2016), though the 3 μm feature would most likely result from contamination by later impacts (Avdellidou et al. 2018).

If Psyche were composed of one of these groups, it might not be the first time a spacecraft has visited such a body. The Rosetta spacecraft flew by asteroid 21 Lutetia, determining a bulk density of $3{,}400\pm300~\text{kg}\,\text{m}^{-3}$ and observed a largely featureless spectra lacking absorption features indicative of either FeO-bearing silicates or hydrated phases (Coradini et al. 2011; Sierks et al. 2011). This high density and lack of obvious absorption features may indicate either an enstatite chondrite composition (Vernazza et al. 2011) or a composition similar to CR, CH, CB and G chondrites (Moyano-Cambero et al. 2016), and in either case Lutetia may be partially differentiated, with a small metal core adding to density (Weiss et al. 2012). Lutetia also has lower radar albedo than Psyche has (Shepard et al. 2015, 2021). In contrast to the limited data available from the Lutetia flyby, the Psyche mission instrument package will provide much greater detail needed to identify meteorite analogs.

### Highly Reduced Material from the Inner Solar System

Irresistibly, with a body like Psyche that appears to have all its iron in the metal phase, the possibility of extremely reduced materials arises. Could Psyche consist of primitive material so reduced that the iron was all driven into the metal phase? Enstatite chondrites might sample such material and, as noted above, have been previously suggested as an analog for (21) Lutetia (Vernazza et al. 2011). The bulk density of 60% of enstatite chondrites measured by Macke (2010) falls within the range of preferred densities for Psyche ($3{,}780\pm340~\text{kg}\,\text{m}^{-3}$). A strength of linking enstatite chondrites and Psyche is the essentially FeO-free nature of the silicates in enstatite chondrites. We note, however, that ubiquitous high-FeO silicates are observed in minimally metamorphosed type 3 enstatite chondrites (Lusby et al. 1987). Given the uncertainties in FeO concentration derived from ground based spectral analyses, enstatite chondrites should be considered as a possible analog (though Psyche’s high radar albedo may be an argument against its being an enstatite chondrite). Weidenschilling (1978) proposed that Mercury might have formed similarly, explaining its large metal fraction, and Kruss and Wurm (2018, 2020) suggest that a concentration of Fe metal-rich particles in the inner solar system may explain the gradation in core sizes of the terrestrial planets. Perhaps Psyche is an analog to that material. As outlined in McCoy et al. (this journal), determination of the redox conditions of Psyche could further confirm or refute such a link.

Kruijer et al. (2017) have shown, however, that iron meteorites are associated with both carbonaceous and non-carbonaceous materials. The Kruss and Wurm (2018, 2020) hypothesized process may not be able to produce CH and CB chondrites beyond Jupiter, where they appear to have originated.

## How We Will Use Our Data to Determine Psyche’s Origin

Discriminating whether Psyche is core material from a differentiated planetesimal, or an unusually metal-rich and FeO-poor chondritic body, will require the combination of measurements and interpretations from all the instruments. Few individual measurements would be definitive on their own; one definitive measurement might be a strong remanent magnetic field, indicating the presence in the deep past of a core dynamo and requiring a differentiated parent.

### Magnetic Field

Magnetometer data may enable discrimination between the competing hypotheses that Psyche formed as the metallic core of a mantle-stripped planetesimal or as an unmelted, iron-rich chondritic aggregate. If Psyche has a strong magnetic moment, this would strongly favor its origin as a mantle-stripped planetesimal. This is because a molten core formed after mantle stripping could generate a long-lived magnetic field by the dynamo process (Bryson et al. 2017; Maurel et al. 2020). The essence of a dynamo is that large-scale advective motions of a conductive fluid can inductively generate currents that amplify an arbitrarily weak initial magnetic field (Moffatt and Dormy 2019). This magnetic field could then be recorded as remanent magnetization by ferromagnetic minerals (e.g., the iron-nickel minerals kamacite and tetrataenite) as they crystallize or cool through their magnetic ordering temperatures on the outside of the cooling core. Because such magnetization can in principle persist for longer than the age of the solar system (Nagy et al. 2019), this remanent magnetization could then be detected by a sufficiently sensitive spacecraft magnetometry investigation. On the other hand, the lack of detection of a remanent field would not exclude origin as a core for several reasons. First, the existence of a molten, advecting, conductive fluid is a necessary but not sufficient requirement for dynamo action: only some flow velocities and geometry will lead to field amplification (Moffatt and Dormy 2019). Second, the iron minerals may not have formed or cooled sufficiently at the time of dynamo generation to record the field.

Alternatively, if Psyche is an iron-rich undifferentiated chondritic body, it would never have melted and therefore generated a dynamo field that could magnetize the body. A potential complication is that the early solar nebula may have generated magnetic fields of similar intensity as dynamo surface fields on iron meteorite parent bodies (Fu et al. 2014; Weiss et al. 2021; Borlina et al. 2021). Recent magnetohydrodynamic simulations indicate this field could have been temporally steady for $>1{,}000~\text{y}$ (Weiss et al. 2021). If a chondritic body were thermally metamorphosed and cooled and/or formed secondary magnetic minerals via aqueous alteration while the nebular field was present, it could have been magnetized by this field. However, this requires that these events occurred on the parent body before the nebular field dissipated. This is challenging because paleomagnetic measurements indicate that the nebular field had already dissipated by 3.9 Ma after solar system formation (Wang et al. 2017; Weiss et al. 2021).

With respect to the nebular magnetization formed as a result of metamorphism, the estimate nebular lifetime is well before the estimated time when ordinary chondrites cooled through kamacite’s 800 C Curie temperature ($\sim5\text{--}60~\text{Ma}$ after solar system formation) (Blackburn et al. 2017). However, it is possible that other metamorphosed chondritic bodies cooled more quickly. With respect to nebular magnetization acquired by secondary alternation minerals, aqueous alteration of many carbonaceous chondrite parent bodies occurred sometime between $\sim2\text{--}11~\text{Ma}$ after solar formation, which encompasses the lifetime of the nebula (Carporzen et al. 2011; Doyle et al. 2015; Jogo et al. 2017). For the secondary magnetic minerals to retain magnetization until the present day, the body must not subsequently heat beyond their unblocking temperatures after the nebular field dissipates. For example, chondritic bodies that form between 3.0 and 3.75 Ma may heat just enough to undergo aqueous alteration before the nebular field dissipates, but not enough to subsequently erase the magnetization (Courville et al. 2022). The solar nebular field is possibly the source of the coherent magnetization observed in the CM and CV chondrites (Cournede et al. 2015; Fu et al. 2021), although a core dynamo is also possible (Carporzen et al. 2011; Gattacceca et al. 2016). In these meteorites, aqueous alteration produced the ferromagnetic iron-oxide and iron sulfide minerals magnetite and pyrrhotite which may retain a record of the magnetic field. Therefore, if Psyche is observed to contain secondary ferromagnetic oxides or sulfides, magnetization by the nebular field should be considered as a possible explanation for some remanent magnetization (Courville et al. 2022). However, this requires a large volume fraction of Psyche (tens of percent or more) to be composed of ferromagnetic oxides and sulfides for the resultant moment of Psyche to be detectable with the magnetometer. In general, the possibility of remanent magnetization from nebular fields on Psyche merits further investigation.

Should magnetic fields be detected by the magnetometer, observations from the GRNS and Imager could potentially distinguish between a chondritic and achondritic surface. Given the relatively young cooling ages of most achondrites and the possibility that their parent bodies formed metallic cores, this will be an important indicator of whether Psyche could have been magnetized by an external source. We can further distinguish between different magnetizing fields from examining the three-dimensional field topology around the body to infer the magnetization pattern. A dynamo-generating core would produce non-uniform magnetization due to the curved geometry of the field (Runcorn 1975) and its likely temporal variation during the gradual solidification of the body (Neufeld et al. 2019). In contrast, a nebular field may impart more uniform magnetization, as the field source is external to the body (Runcorn 1975). These two types of magnetization configurations could be distinguished using a combination of data analysis and modeling tools developed by the Magnetometry Investigation (Weiss et al. this journal).

Magnetometer data can also be used to constrain the bulk electrical conductivity of Psyche by detecting induction fields due to variations of the solar wind that Psyche is exposed to. If the body has very low bulk conductivity the induced fields outside the body would be weak (a Moon-like interaction); if highly conducting, there would be a region of enhanced magnetic field in the upwind side of the body (a Venus-like interaction). The value of resistivity producing each type or reaction depends on the solar wind speed and the body size (Oran et al. 2018). A body the size of Psyche and a solar wind speed of $400~\text{km}\,\text{s}^{-1}$ would behave as a perfect resistor for resistivity $\eta \gg 4\times 10^{4}~\Omega \text{m}$ and as a perfect conductor for $\eta \ll 4\times 10^{4}~\Omega \text{m}$. Chondrites have a resistivity of $10^{5}~\Omega \text{m}$ (Duba and Boland 1984), rocky achondrites materials like the lunar crust can have resistivities near $10^{8}~\Omega \text{m}$ (Dyal et al. 1977) while an iron metal-rich body would have a resistivity of iron of $10^{7}~\Omega \text{m}$ (Secco and Schloessin 1989). However, if the body contains a thick outer layer with high resistivity, a more conducting interior would be masked (Oran et al. 2018). Further, if the body is porous this would reduce its bulk resistivity.

### Scale of Silicate/Metal Mixing

If Psyche is the stripped remnant of a differentiated planetesimal, its metal and non-metal fractions should exist in meter- to kilometer-scale or larger regions. If, alternatively, its bulk composition is chondritic, its metal and non-metal will be intimately mixed on a millimetric or centimetric scale.

For meteoritic materials, the metal-to-silicate fraction as determined by laboratory methods like XRF spectroscopy is typically defined as the total amount of metal (e.g. metallic iron, nickel) versus total silicates (elements bound as oxides). In that scenario, iron in silicates (e.g. FeO) is “book kept” with silicates. In contrast, gamma-ray and neutron spectroscopy measures elements without regard to their mineralogical state. Since the largest concentration of “metal” elements being considered for Psyche consist of Fe and Ni, for the purpose of GRNS measurements we define the “metal” content as the total concentration of Fe and Ni. “Silicates” are then defined as other major rock-forming elements (Si, O, Al, Mg, Ca, etc.).

The highest sensitivity GRNS metal-to-silicate measurements are accomplished using neutron data from the Neutron Spectrometer (NS). The NS measures neutrons in three energy bands; thermal, low-energy epithermal, and high-energy epithermal neutrons. The Gamma-Ray Spectrometer (GRS) anti-coincidence (AC) shield provides a measure of fast neutrons (Lawrence et al. 2019, 2016). Thermal neutrons and fast neutrons are independently sensitive to the concentration of Fe and Ni via their thermal-neutron absorption properties (e.g., Feldman et al. 1998; Peplowski et al. 2016) and average atomic mass (e.g., Gasnault et al. 2001; Lawrence et al. 2017), respectively. High metal fractions result in relatively low thermal neutrons and high fast neutrons; the converse is true for high silicate fractions. On Psyche, it is expected there will be variations in H concentrations that affect the neutron measurements (Reddy et al. 2018). To account for such effects, Lawrence et al. (this journal) developed an analysis framework based on principal component analysis that enables the four Psyche neutron measurements to uniquely discriminate the three compositional parameters of metal-to-silicate fraction, H concentration, and relative Ni variations.

The spatial scale of the measured metal-to-silicate fraction is determined by the altitude of the spacecraft of Psyche’s surface, and the full-width at half-maximum of the footprint can be approximated as $\sim1$ to 1.5 times the altitude (Lawrence et al. 2003). For the lowest orbit of the mission, altitude is required to be less than one Psyche radius ($R_{P}$) and is targeting an altitude of 0.8 $R_{P}$ (Polanskey this journal). This altitude corresponds to an effective spatial footprint of $\sim100$ to 150 km, which is a spatial scale of a large “province” on Psyche; this footprint may be reduced using spatial deconvolution techniques (Wilson et al. 2018). In a practical sense, the spatial resolution of the metal-to-silicate measurement will be similar to the gamma-ray and neutron measurements achieved with the Dawn mission at the asteroids Vesta and Ceres (Prettyman et al. 2017, 2013). Although the presence and spatial scale of any geochemically-distinct regions on Psyche are unknown, there is evidence for hemispherical-scale variations in optical albedo (Takir et al. 2016) and large-scale radar albedo (Shepard et al. 2021) that would be spatially resolvable by GRNS if they correspond to chemical differences.

The scenario is somewhat different, however, for the metal-to-silicate fraction inferred from visible to short-wave near-IR ($\sim400$ to 1,100 nm) multispectral imaging or spectroscopy. Specifically, the presence of an optically-significant metallic component can be inferred from an assessment of the slope of the reflectance spectrum across this full wavelength range, with the reddest slopes being consistent with the presence of a significant metallic iron and/or iron-nickel component, based on laboratory studies of iron meteorites. In contrast, specific relatively broad solid-state absorption features within this wavelength range are used to infer the presence of certain iron-bearing silicates known to be important components of chondritic meteorites and certain common classes of asteroids (like the S-types, which can exhibit diagnostic absorption features indicating the presence of olivines and/or pyroxenes).

Unlike GRNS or laboratory elemental techniques, however, multispectral observations in the visible to short-wave near-IR cannot typically be used to infer absolute elemental or mineralogic abundances. This is because of a variety of fundamental ambiguities in the interpretation and modeling of such data caused by the competing effects of particle size, particle shape, degree of compaction, viewing geometry, and other factors. Thus, multispectral observations like those to be made with the Psyche Multispectral Imager can be used to *detect* metallic iron- and nickel-bearing materials as well as to *detect* the presence of some common rock-forming iron-bearing silicates, but abundances of these phases can usually only be inferred or modeled in a relative sense. Still, the limitations on deriving absolute abundances can be offset to some degree by the ability to map relative variations in the inferred metal-to-silicate ratio at high spatial resolution across a planetary surface. As well, correlations between the imaging-based detections of such surface compositional/mineralogic components and other measured surface properties (geologic context, topography, magnetic field strength, elemental composition, etc.) can provide important additional constraints to factor in to multispectral data interpretation. Thus, each of the Psyche mission’s different elemental/mineralogic measurement techniques provides a different and complementary perspective on the composition and mineralogy of the asteroid.

### Correlation of Gravity and Composition

If Psyche’s current internal structure predominantly reflects solidification from a liquid state, its interior would likely have a radially-distributed density structure overlain by a layer of surface materials presumably pulverized by impacts and containing exogenic material. In contrast, if Psyche was significantly disrupted and/or was never molten, it would likely have an azimuthally-varying or heterogeneous density structure at depths greater than its lid. The correlation between Psyche’s gravity field with its shape could be indicative of structure and degree of spherical symmetry or breakage into rubble (Konopliv et al. 2014; RS Park et al. 2014, 2016, 2020). Furthermore, the relative amplitudes of gravity and topography over a range of wavelengths (as quantified by the admittance) will be sensitive to the density of surface materials and how density varies with depth below the surface. For example, the correlation of Vesta’s gravity field with an assumed three-layer model is approximately 1 for all measurable degrees (Konopliv et al. 2014; Park et al. 2014), indicating no substantial density variations in Vesta’s crust, nor a strong contrast in density between the assumed crustal layer and the mantle. Such a high correlation could also be expected for impact-related homogenization (Zuber et al. 2013). The Psyche gravity science investigation would recover a degree 10 gravity field (Zuber et al. this journal), which would allow discriminating density heterogeneity at the scale of $\sim1/10$ of Psyche.

### Silicate Compositions

GRNS measurements of the elemental composition of silicates are primarily measured using gamma-ray data, and supplemented using neutron data. For GRNS data it is important to note that the gamma-rays and neutrons are sensitive to Psyche’s bulk composition and therefore do not and cannot distinguish between the surface’s mineralogic form or mixture. The primary elements used to characterize Psyche’s silicate compositions are Fe, Si, Al, K, and Ca. As described in Elkins-Tanton et al. (2020), various combinations of these elements can discriminate the type of material (chondritic versus achondritic) that is present on Psyche. The specific detection sensitivities for these elements are given in Lawrence et al. (this journal).

The Imager’s study of silicates will be primarily based on the detection, characterization, and mapping of the so-called “1-micron” absorption band in iron-bearing silicates like pyroxene and olivine (see, e.g., reviews in Burns 1993; Gaffey et al. 1993; Bishop 2020). Specifically, the required signal to noise ratio in the Imager’s four longest-wavelength near-IR filters (at 725, 850, 948, and 1042 nm; Bell et al. this journal) will provide the ability to detect weak (≥ around 1% band depth) absorption in the 1-micron region potentially indicative of the presence of specific iron bearing silicates. For example, a weak feature near 900 nm has been reported by some observers in whole-disk telescopic spectra of Psyche and has been interpreted as evidence for a silicate component – potentially low-Fe pyroxene (e.g., Clark et al. 2004; Hardersen et al. 2011, 2005). Additionally, laboratory studies of metal/silicate mixtures show that the relative abundances of the kinds of iron-bearing silicate components that could reasonably be hypothesized on the surface of Psyche based on telescopic data and meteorite analogs could indeed yield very weak absorption features. For example, Cloutis et al. (2009) showed that 90:10 mixtures of metal and olivine produce an absorption feature in the 1-micron region that is only $\sim1\%$ deep. The Imager will search for such weak absorptions by calculating (and mapping) ratios of observed surface reflectances (e.g., between 725 nm and the three longest wavelength filters), band depth maps (using 725 nm and 1042 nm images as “continuum” points), and correlations between such derived ratios and band depths with other parameters, such as 1-micron band area and/or overall albedo (e.g., Dibb 2021).

### High S, K on Surface

High concentrations of sulfur and possibly potassium on the surface of Psyche would be indicative of ferrovolcanism. The immiscible, sulfur-rich liquid produced by fractional solidification of a metal core is expected to contain approximately 50 mol% sulfur (it is effectively liquid troilite; Jones and Drake 1983), and other incompatible elements like potassium may also be present. This liquid is a candidate for expulsion onto the surface of a solidifying differentiated planetesimal.

The GRNS is sensitive to K and S with gamma-ray data. K is naturally radioactive and produces a relatively high flux of gamma rays, allowing highly sensitive measurements of K abundances down to 0.02 wt.% (or 200 ppm). Numerous examples exist of K measurements at other planetary bodies using orbital gamma-ray data (e.g., Lawrence et al. 1998; Peplowski et al. 2011; Prettyman et al. 2015). The GRNS will provide a K map (to the sensitively limit of 0.02 wt%) with a spatial resolution similar to the neutron data. Thus, locations of enhanced K abundances should be detectable by GRS data. S concentrations greater than 3 wt.% can also be detected with GRS, and this capability has been demonstrated with other high-purity germanium sensors at Mars and Mercury (King and McLennon 2010; Evans et al. 2012).

The Imager provides sensitivity to S only via the search for and potential detection of specific sulfides that exhibit a diagnostic absorption feature in the visible wavelength region, such as oldhamite ((Ca,Mg,Fe)S). Specifically, the Imager will use narrowband filters at 437, 495, and 550 nm to detect and map a weak cubic monosulfide feature in sulfides like oldhamite, which has been identified in laboratory and telescopic data at these wavelengths (e.g., Burbine 2014; Burbine et al. 2002a,b; Clark et al. 2004; Gaffey et al. 1993). Ratios among these filters and band depth maps at 495 nm using 437 nm and 550 nm as the continuum, will be the primary derived products used in the imaging search for signatures on Psyche of S-bearing phases with local mixture abundances greater that around 10%.

### Nickel Content

The nickel content of the metal phase is indicative of the crystallization sequence of the core, if such it is, and of the oxidation or reduction state of the material, in the case that it is not a core. The earliest solids fractionally solidifying from a liquid core are the lowest in nickel content, and the last solidifying metal crystals contain the highest nickel content. Ni abundances are primarily measured with the 1.454 MeV gamma-ray line, however Ni produces additional line signatures that can be used to characterize Ni abundances (albeit with lower sensitivity than the 1.454 MeV line) (Peplowski et al. 2019). GRS data will measure Ni where present at concentration of 4 wt.% or higher (Lawrence et al. this journal), averaged over the spatial resolution of the GRNS measurements. In addition to the gamma-ray measurements, combined neutron data will be sensitive to relative Ni variations across Psyche’s surface should they be present (Lawrence et al. 2021). Thus, GRNS measurements provide a robust means for determining Psyche’s Ni concentration, which is a key measurement for understanding Psyche’s origin.

### Combining Instrument Measurements to Reach Conclusions

**Fig. 6:**
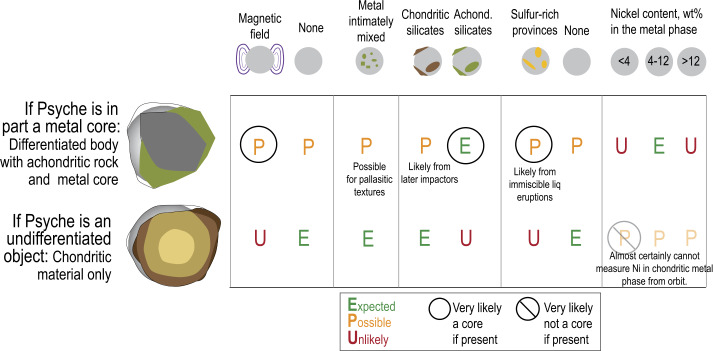
This matrix of instrument measurement interpretations along the top shows the requirement for multiple measurements to support either of the origin hypotheses at left. Only a few measurements are probably definitive on their own (strong magnetic field, achondritic silicate provinces, and sulfur-rich volcanism each likely indicate Psyche is a core). Expected = Likeliest outcome according to current models; Possible = Outcome predicted in some scenarios; Unlikely = Not predicted in current models. See text for detailed explanation, and for further explanation of how the instruments can discriminate between achondritic and chondritic silicates, see Elkins-Tanton et al. (2020)

The requirement to combine many instrument observations to answer the question of Psyche’s origin is shown in Fig. 6. If there is a strong magnetic field, we can conclude that Psyche is a part of a differentiated body. The scale of metal and silicate mixing is a second key observation. If Psyche’s materials contain intimately mixed metal and silicate (on a millimetric or centimetric scale, as in chondritic meteorites), then the most likely interpretation, based upon the meteorite collection, is that the material is primordial and unmelted. However, intimate mixing of silicates and metals is also characteristic of the less common pallasitic meteorites, and so intimate metal-silicate mixing is not alone a definitive measurement.

Silicate and metal provinces may also exist on a macroscopic scales, even up to tens to hundreds of kilometers. Regions of chondritic material lying on top of metal are almost certainly the result of later impactors, that is, the chondritic material is exogenous to the Psyche parent body. Achondritic impacts are far less common, judging from both asteroid spectra and meteorite incidences. Non-chondritic silicate patches would thus likely be patches of the silicate fraction of Psyche’s parent body and strongly indicate that Psyche is a part of a differentiated parent body.

The fractional solidification of metal cores almost inevitably produces an immiscible liquid (Ulff-Muller 1998; Chabot 2004; Goldstein et al. 2009) with the approximate stoichiometric composition of a troilite-like monosulfide: [(Fe,Ni)_1 − *x*_S] (Jones and Drake 1983). This immiscible liquid is less dense than its coexisting more Fe-rich liquid core, and so it is expected to rise to the surface or potentially to erupt through the contracting solid lid of a core freezing from the outside inward (Abrahams and Nimmo 2019; Elkins-Tanton et al. 2020). Large provinces of sulfur-rich material on Psyche’s surface, therefore, would indicate that Psyche’s parent body was differentiated; there is no obvious mechanism by which to make strongly sulfur-enriched regions on a chondritic parent body.

When metal cores fractionally solidify, the earliest-forming solids have a low nickel content, around 4 wt%. Most iron meteorites have nickel contents between 4 and 12 wt% (Buchwald 1975; Wasson et al. 2007 and references therein; Goldstein et al. 2009; Scott 2020). Above 12 wt% nickel in the metal fraction probably indicates that the parent body may have been highly oxidized (driving iron into the mantle and effectively raising the nickel fraction in the core), though IVB iron meteorites have more than 12 wt% nickel (e.g. Rasmussen et al. 1984; Goldstein et al. 2009) and they are believed to have formed in a core. (Some iron meteorites, suspected to have come from highly oxidized parent bodies, contain over 30 wt% and as much as 60 wt% nickel; these include Tishomingo and Dermbach [Buchwald 1975], and the IAB-ungrouped Oktibbeha County [Wasson and Kallemeyn 2002]).

In summary, if Psyche has a strong magnetic field, then it is almost certainly from a differentiated parent body. Similarly, large achondritic rock provinces on its surface are most likely to be remnants of its original mantle, and thus Psyche is a mixture of core material and mantle. Sulfur-rich (S) volcanism is only expected for a differentiated body. Nickel content in the metal phase below 4 wt%, however, indicates a highly oxidizing environment and is lower than iron meteorite analogs, and thus Psyche is not likely to be a core, in the absence of contradictory evidence. All other measurements need to be considered together in creating conclusions about Psyche’s provenance.

## Conclusions

The bulk density of asteroid (16) Psyche appears to be between 3,400 and $4{,}100~\text{kg}\,\text{m}^{-3}$. Given the known densities of meteoritic metal, and assuming not more than 20% porosity, the body is thus predicted to contain up to 60 vol.% metal. If porosities higher than expected on a body of Psyche’s size are enabled by the material properties of metals at relevant space temperatures, then Psyche’s metal fraction can be far higher than 60 vol%. Indeed, in the most extreme case, the bulk density is consistent with pure iron-metal with $\sim50\%$ porosity.

The asteroid is therefore likely a mixture of metal and an additional lower-density component or components. Based upon modeling of available telescopic and laboratory spectral characteristics, the additional components are likely to be very low in FeO or Fe_2_O_3_ (Dibb 2021). Likely candidates for a non-metal fraction of the asteroid include low-iron pyroxene (e.g., Hardersen et al. 2005), sulfides (e.g., Clark et al. 2004), or perhaps carbon compounds. By constraining the composition and density of the non-metal component, it will be possible to place tighter bounds on the metal volume fraction of Psyche.

Our currently favored fiducial model for the asteroid’s formation is that it is the remains of a differentiated body containing core metal. Psyche may contain a large fraction of a metal core and some silicate mantle retained on the outside, or the silicate fraction could be mixed into the metal fraction. The silicate fraction must, however, be strikingly low in iron oxide.

Formation of such a low iron-oxide bulk rock composition seems, within the current state of planet formation theory, to require one of two processes to have occurred: (1) Silicate differentiation that produces low-iron-oxide silicates near the core and high-iron-oxides silicates nearer the surface, with subsequent removal of the outermost high-iron-oxide fraction by stripping impacts; or (2) Formation of the Psyche parent body from highly reduced primitive material in which the iron has been reduced to its metallic form and incorporated into the core, leaving the silicates naturally low in iron and high in magnesium.

Differentiation of a parent body followed by mantle stripping may be the simplest model to call upon to form a metal-enriched Psyche. Stripping the parent body to reduce the low-density phase and reveal metal from the core can be done via erosion by multiple impacts, by removing a portion of the deep mantle of a larger body via one catastrophic impact, or by stripping the mantle off the impactor during a hit-and-run collision. Erosion by multiple impacts may be impossible to reconcile with the preservation of the basaltic crust of Vesta. Blasting apart in a single catastrophic impact is also challenging because the parent body, being larger than Psyche, might be too large to destroy after the Main Belt was emplaced. A hit-and-run disruption seems most likely, although this would have to happen during planetesimal accretion, in the first few million years.

The parent body could be a differentiated inner-solar-system or outer-solar-system planetesimal, with different implications for Psyche’s composition. The semimajor axis distribution of M-types appears different from planetesimals expected to have come from the inner solar system, and more consistent with an outer solar system origin. If so, then disruption by a hit-and-run event would have happened when interactions among similar-sized planetesimals were more common, before or during scattering and implantation in the Main Belt.

The deep interior of an outer-solar-system parent body may be volatile rich, depending on its early thermal evolution, with hydrated phases in the mantle and possibly volatiles sequestered in its core. Removal of the mantle (by whatever means) would expose these phases to low pressure, in which case a subsequent early epoch of sublimation-degradation or volatile eruption may have occurred. This might not be the case for an inner-solar-system parent body, whose deep interior may be dunitic rock grading towards metallic core-like compositions, that would be stable at lower pressures.

As an alternative to these disruption scenarios, Psyche might be an undifferentiated body consisting of a highly reduced, low-FeO material. Meteorite analog candidates include enstatite chondrite or CR, CH, or CB chondrites, some example of each of which have adequately low FeO and high density.

The Psyche mission project will determine Psyche’s origin and formation of the asteroid by measuring any strong remanent magnetic field, which would imply it was the core of a differentiated body; the scale of metal to silicate mixing will be determined by both the neutron spectrometers and the filtered images; the degree of disruption between metal and rock may be determined by the correlation of gravity with composition; some mineralogy will be detected using filtered images; and the nickel content of Psyche’s metal phase will be measured using the GRNS.

## Data Availability

All data and material can be found in cited material; no original data was generated for this paper.
